# Real Time Forecasting of Measles Using Generation-dependent Mathematical Model in Japan, 2018

**DOI:** 10.1371/currents.outbreaks.3cc277d133e2d6078912800748dbb492

**Published:** 2018-10-15

**Authors:** Andrei R. Akhmetzhanov, Hyojung Lee, Sung-mok Jung, Ryo Kinoshita, Kazuki Shimizu, Keita Yoshii, Hiroshi Nishiura

**Affiliations:** Hokkaido University; Hokkaido University; Hokkaido Univeristity; Hokkaido University; Hokkaido University; Hokkaido University; Graduate School of MedicineHokkaido University

**Keywords:** Forecasting, Measles

## Abstract

Background: Japan experienced a multi-generation outbreak of measles from March to May, 2018. The present study aimed to capture the transmission dynamics of measles by employing a simple mathematical model, and also forecast the future incidence of cases.

Methods: Epidemiological data that consist of the date of illness onset and the date of laboratory confirmation were analysed. A functional model that captures the generation-dependent growth patterns of cases was employed, while accounting for the time delay from illness onset to diagnosis.

Results: As long as the number of generations is correctly captured, the model yielded a valid forecast of measles cases, explicitly addressing the reporting delay. Except for the first generation, the effective reproduction number was estimated by generation, assisting evaluation of public health control programs.

Conclusions: The variance of the generation time is relatively limited compared with the mean for measles, and thus, the proposed model was able to identify the generation-dependent dynamics accurately during the early phase of the epidemic. Model comparison indicated the most likely number of generations, allowing us to assess how effective public health interventions would successfully prevent the secondary transmission.

## Introduction

Measles is a highly contagious viral infectious disease transmitted by aerosol 1. Clinical symptoms of measles include fever, sore throat, conjunctivitis, and rash, and it can potentially be lethal to infants and children, leading to serious complications including encephalitis and neurological complications 2. In Japan, since the year 1978, routine vaccination against measles have started, and a two-dose regimen has been introduced among birth-cohorts born in and after 1990, contributing to reducing the burden of measles by elevating the immunity level in the population 3^,^^4^. The transmission of the virus in Japan has not been sustained, and the Measles Regional Verification Commission of the World Health Organization (WHO) Regional Office for the Western Pacific verified Japan as having achieved measles elimination in March 2015 5. However, global circulation of the virus continues to pose a risk of sporadic outbreaks to Japan 6.

From March 2018, an abrupt outbreak in Okinawa has been notified ahead of the “Golden Week”, the longest vacation period of the year (i.e., from 28 April to 6 May 2018). The index case was a 30-year-old Taiwanese man who had a travel history to Thailand in early March. On 17 March 2018, he flew to Okinawa, and on the third day of his stay in Okinawa, he sought for medical service. Following an incubation period of 11-12 days after the diagnosis of the index case 7, multiple generations of local cases were identified in Okinawa prefecture. Cases originating from Okinawa prefecture produced multiple chains of transmission, bringing a total number of confirmed cases to 124. The spread could not have been contained within Okinawa, and spread to Aichi prefecture, Kanagawa prefecture, and Tokyo Metropolis.

During this outbreak, measles cases have been confirmed at governmental diagnostic research facilities and reported in real-time. Each report was regarded as a snapshot of the growing epidemic curve that was used for forecasting of the future course of the outbreak. To understand better the transmission dynamics during the course of an outbreak, we implemented the future forecast to infer public health control activities. While not explicitly assessing the control activities in our exposition, the purpose of the present study is to capture the transmission dynamics of measles by employing a simple parsimonious mathematical model and to forecast future generations of measles incidence.

## Methods

*Epidemiological data*

Measles is clinically diagnosed by the presence of a generalized rash, fever, and catarrh symptoms, such as cough, coryza, or conjunctivitis, and then laboratory confirmed. The laboratory confirmation is performed by detection of measles-specific immunoglobulin M (IgM) antibodies 8 or real-time reverse transcription polymerase chain reaction (rRT-PCR). There is an additional clinical form of measles, “modified measles”, that usually exhibits only one of three symptoms, is laboratory confirmed, and has a milder clinical course of illness. The present study rests on governmental reports based on outbreak investigation in Japan 9, including local governmental reports from prefectures with at least one case, i.e., Okinawa, Aichi, Kanagawa and Tokyo Metropolis 10^,^^11^^,^^12^^,^^13^. We retrospectively scanned all real-time reports of the outbreak and reconstructed the epidemiological dynamics of measles that developed from the identical case. Dates of illness onset and laboratory confirmation, retrieved from those governmental reports, allowed us to characterize the epidemic dynamically evolving in time.

*Inference procedure*

Due to close contact tracing practice, we assumed that all cases were certainly diagnosed and reported. To quantify the underlying epidemiological dynamics and delay distribution from illness onset to laboratory confirmation, we employed the maximum likelihood estimation technique. Specifically, we considered the total (composite) likelihood function *L_Σ_* consisting of two parts, each corresponding to different pieces of the dataset, i.e., (i) individual datasets of the dates of illness onset and laboratory confirmation, and (ii) the number of new cases by the date of illness onset. The first part allowed us to identify the distribution of the time delay that was subsequently used to predict the number of cases who have yet to be confirmed. We introduced a probability density function *h*(*d_n_*; ***θ**_h_*) that measured the probability of the case *n* = {1 … *N*} to be confirmed on *d_n_* days after the onset of symptoms. We assumed that the distribution *h* does not vary as a function of calendar time during the course of the outbreak. An alternative formulation of a time-varying distribution *h* did not improve the model fit and hence was discarded here (see Appendix A). Using the first part of the dataset, we arrived at the likelihood function of the form:

\begin{equation*}L_{\text{delay}}(\mathbf{\theta}_h;\,d_n)=\prod\limits_{n=1}^N
                        h(d_n;
                    \mathbf{\theta}_h).\end{equation*}

We assumed that *h* follows a Weibull distribution with parameters the mean and variance *v_h_* (***θ**_h_* = {*μ_h_*, *v_h_*}). The second part of the likelihood utilizes a part (ii) of the dataset that describes the incidence on a day *t* = {1 … *T*} denoted as *i_t_*. The incidence follows a Poisson distribution:

\begin{equation*}L_{\text{onset}}(\{\mathbf{\theta}_\Lambda,\mathbf{\theta}_h\};\,i_t)
                        = {}\end{equation*}

\begin{equation*}\prod\limits_{t=1}^T\frac{(H(T-t;\,\mathbf{\theta}_h)\cdot\Lambda_t(\mathbf{\theta}_\Lambda))^{i_t}\exp({-}H(T-t;\,\mathbf{\theta}_h)\cdot\Lambda_t(\mathbf{\theta}_\Lambda))}{i_t!}\,,\end{equation*}

where *H* is a cumulative distribution of the delay *h*, counted backwards in time from the day of the update publication *T*.

The incidence function Λ*_t_*(***θ***_Λ_) is modeled by sequential generation process (hereafter, referred to as the generation-dependent model). Each new case has an ability to generate new secondary infections with the probability density function of the generation time *g_t_*. In brief, we describe this by the following epidemiologic process. The index case solely belongs to the first generation. It first generates new *R*_1_ cases distributed in time according to the distribution *g_t_* that constitute the second generation. Each secondary case subsequently generates *R*_2_ tertiary cases according to the same distribution *g_t_* resulting in the third generation. Because of independently and identically occurring transmission events, the total number of cases at calendar time *t* is given by the formula: *R*_1_(*g_t_* + *R*_2_(*g*∗*g*)*_t_*), where the symbol “∗” stands for the convolution operator of two functions on its left- and right-hand sides. Specifically, a convolution operator of the functions *f*_1_ and *f*_2_ at time *t* yields the following formula:

\begin{equation*}(f_1\ast f_2)_t =
                        \sum\limits_{\tau=1}^{t-1}f_1(t-\tau)\cdot
                        f_2(\tau),\end{equation*}

where *f*_2_(0) is equal zero. The above mentioned method is restricted to three generations, however, we can assume any arbitrary number of new generations for describing an epidemic curve. For example, if we account for up to four generations, the total number of cases is written accordingly as: *R*_1_(*g*_*t*_ + *R*_2_((*g*∗*g*)*_t_* + *R*_3_(*g*∗*g*∗*g*)*_t_*)).

However, the rate Λ_*t*_ needs to be normalized to the expected cumulative number of all cases *K*, which means:

\begin{equation*}\Lambda_t(\mathbf{\theta}_\Lambda=\{K,R_{1,2,3}\})=K\frac{R_1g_t+R_1R_2(g\ast
                        g)_t+R_1R_2R_3(g\ast g\ast
                        g)_t}{N_R},\end{equation*}

where *R_m_* is the effective reproduction number of the (*m* + 1)-th generation, *N_R_* represents a normalization constant restricting the total number of cases to *K*. Hence, *N*_*R*_ = *R*_1_ + *R*_1_*R*_2_ + *R*_1_*R*_2_*R*_3_. See Appendix B for the derivation of generation-based model. Due to the normalization, the parameter *R*_1_ cannot be recovered, only its lower bound can be identified by manually counting the number of secondary cases who can certainly identified as caused by the index case. The model fit for shorter time horizon of forecasting may require a smaller number of generations, and thus, the latest formula would need to be modified (e.g. *R*_3_ = 0 in case of three generations only, and *R*_2_ = *R*_3_ = 0 in case of two generations only, governing the entire dynamics of the observed epidemic data). As for the generation time distribution *g_t_*, we employ a gamma distribution with the mean 11.7 days and variance 9.0 day^2^ (the average of two previously reported estimates 7).

The total likelihood is:

\begin{equation*}L_\Sigma(\mathbf{\theta}=\{\mathbf{\theta}_\Lambda,\mathbf{\theta}_h\};d_n,i_t)=L_{\mathrm{delay}}(\mathbf{\theta}_h;d_n)\times
                        L_{\mathrm{onset}}(\{\mathbf{\theta}_\Lambda,\mathbf{\theta}_h\};i_t)\,,\end{equation*}

subject to maximization with respect to five parameters (***θ*** = {*K*, *R*_2_, *R*_3_, *μ_h_*, *v_h_*}). By using an equivalent minimization of the negative logarithm of the likelihood, we gain the optimal parameter values ***θ*** = ***θ***_0_ as well as the Hessian matrix *H*(***θ***_0_). To reconstruct the confidence intervals and the compute the prediction interval, we implement the matrix *H* into the parametric bootstrapping. First, we design a dataset that consists of model parameters sampled from the normal distribution with the mean ***θ***_0_**,** and standard deviation ***σ*** equal to the square root of diagonal elements of the inverse Hessian matrix (***σ***^2^ = diag(*H*^-1^(***θ***_0_))). Then for each identical set of parameters, we obtain a possible variation in estimated parameter values. Finally, by taking 2.5th and 97.5th percentile points of the simulated distributions, we obtain 95% prediction intervals of incidence function.

*Forecasting procedure*

To perform forecasting exercise, we used an epidemic curve of new measles cases, routinely collected and updated every eight days. As a result, we obtained multiple snapshots of the epidemic curve, all initiated with the date of exposure to the index case on 17 March 2018, but constrained by the date of publication (ranged from 1 April to 25 May with a time step of eight days). Data points of each epidemic curve were then imputed to our model to identify expected number of cases over the time interval of that epidemic curve. Furthermore, the cases were forecasted for an extended time period, up until 8 June.

## Results

The number of new cases of measles by the date of illness of onset and date of laboratory conformation are shown as Figures 1A and 1B, respectively. As of 21 August 2018, a total of 124 laboratory confirmed cases have been reported in Japan, of which 99 cases have been in Okinawa, 23 in Aichi, 1 in Kanagawa prefecture, and 1 in Tokyo.

**Date of illness onset and laboratory confirmation of reported measles cases in Japan, March-May, 2018.**
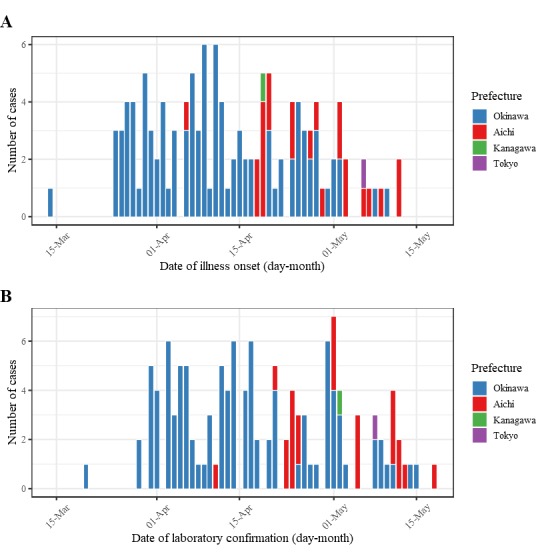
(A) Date of illness onset of measles cases reported in Okinawa, Aichi, Kanagawa prefectures, and Tokyo Metropolis, Japan. Illness onset was unknown for 6 cases notified in Okinawa prefecture, thus, was assumed to be 5 days before laboratory confirmation. (B) Date of laboratory confirmation of measles cases reported in Okinawa, Aichi, Kanagawa prefectures, and Tokyo Metropolis.

Using the observed epidemiological data from Okinawa, Aichi, Kanagawa, and Tokyo reported from 1 April to 25 May 2018, unknown parameters were estimated as shown in Figure 2. In addition, a penalized likelihood for the models of different number of generations was compared based on Akaike Information Criterion (AIC). The minimal value of AIC was used to determine the best-fit number of generations for a given date of publication of the dataset.

The course of outbreak observed before 9 April fitted well using only two generations. Afterwards, the third generation was identified, and the fourth generation appeared since 25 April. The effective reproduction number of the third generation *R*_2_ became greater than one on 9 April. When a new generation was identified to better explain the observed incidence pattern, the model with greater number of generations fitted better than the model with fewer generations. Importantly, our model explicitly accounted for the time delay from illness onset to diagnosis, and thus the effective reproduction number of the most recent generation avoided serious underestimation. However, the expected value of *R*_4_ during the early stage was smaller than during the later stage – the number of cases in the fifth generation was not substantial in May, and thus *R*_4_ was accompanied by a wider confidence interval.

**Estimated parameters values and model comparison by the epidemic date of forecasting.**
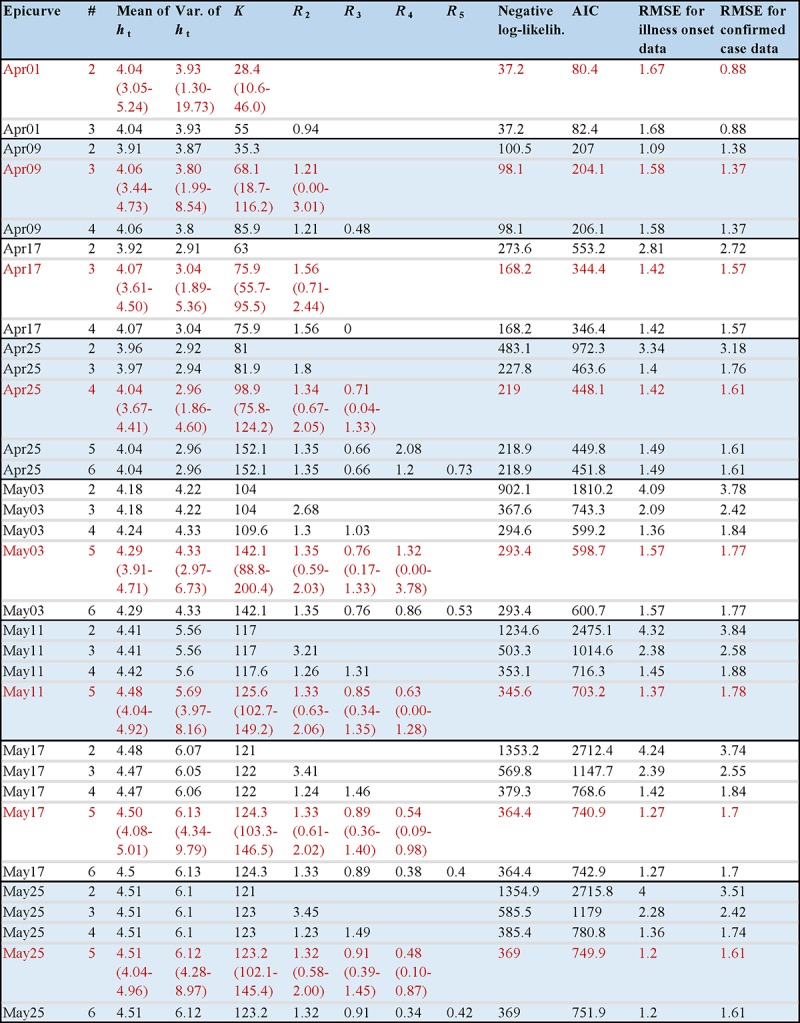
"#" denotes the assumed number of generations in the model. ht is the probability mass function from the time of illness onset to laboratory confirmation. *R_m_* is the reproduction number of (*m* + 1)-th generation. AIC is Akaike information criterion. RMSE is the root-mean-square error. *K* is the estimated total number of symptomatic cases in Japan. Selected models with minimal AIC are shown in red. 95% confidence intervals (CIs) for each model parameter are shown in brackets.

Using the latest snapshot of the epidemic curve published on 25 May, the mean delay from illness onset to confirmation was 4.5 days (95% CIs: 4.0-5.0), and the variance was 6.1 day^2^ (95% CIs: 4.3-9.0). The total number of symptomatic cases *K* which was unknown on the date of publication as some cases with symptoms could still undertake laboratory identification, was estimated as 123.2 (95% CIs: 102.1-145.4). The obtained estimate was close to the observed total number of 123 cases, excluding the index case.

Figure 3 shows the forecasted course of the measles epidemic by using the proposed generation-dependent mathematical model, and the data of confirmed cases of each epidemic curve according to different confirmed date. In the first stage, the model describes only the initial part of the outbreak, but the estimates become certainly improved and the 95% prediction intervals progressively become narrower as more data are used.

**Real time forecasting result of measles in Japan, 2018.**
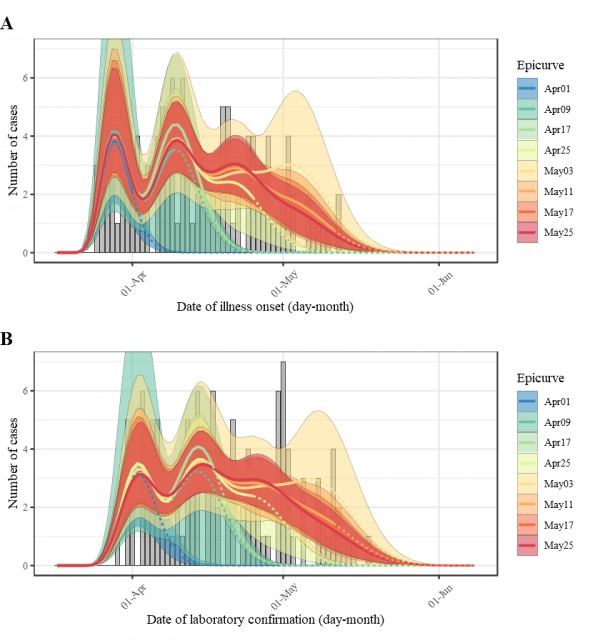
Performance of forecasting for each epicurve (legend) is compared to the number of reported cases in the latest update (bar chart in grey) by date of illness onset of measles cases (A) and date of laboratory confirmation of measles cases (B). Dashed lines denote the forecasting part for each snapshot of the epicurve.

The following Video available online (Figure 4, doi:10.6084/m9.figshare.6991367) presents an extended version of Figure 3 with daily snapshots of the epicurves. As we see, there is a greater degree of uncertainty in the future forecast once a new generation of cases appears and is accounted in the model.

**Animated real time forecasting result of measles in Japan, 2018, with daily snapshots of epicurves.** 

## Discussion

The present study tackled real-time forecasting of measles, employing a generation-specific modelling approach. A simple functional model with generation structure was employed, and the time delay from illness onset to diagnosis was explicitly taken into account. The proposed model helped not only to forecast the future incidence but also to obtain the generation-specific estimates of the effective reproduction number. AIC values helped to identify the most likely number of generations in real-time, allowing us to assess how good public health interventions successfully prevented transmission events during the outbreak. To our knowledge, the present study is the first study to apply the functional generation-dependent model to the context of real-time forecasting.

There are two take home messages. First, the generation-dependent mathematical model successfully helped to anticipate the likely size of the future epidemic in real time. Because the variance of the generation time for measles is relatively limited compared to the mean, the generation-specific number of cases was even manually identified during the early phase of the outbreak 14. This was consequently used in the model. Nevertheless, the reliance on the number of generations can also be regarded as a disadvantage – the model is unable to forecast future generations without appearance of a cluster of likely new cases from the next generation in empirical data or without imposing strong assumptions, e.g., that the effective reproduction number remains the same for a series of the next generations. Thus, we may regard our model as yielding the real-time forecast only for a minimal bound of the future incidence.

Second, the estimation of the effective reproduction number as the weight for the mixture distribution of the generation time is also a side-product of the model (see Appendix B). Without doubt, the reproduction number helps to evaluate preventive measures during the outbreak. Nevertheless, our study also addressed a possible underestimation of the effective reproduction number for the latest generation once considering an explicit time delay from illness onset to laboratory confirmation. Although we did not incorporate stochasticity in the functional model, our model was able to capture the mechanistic pattern of the transmission dynamics.

Few technical limitations must be described. First, the absence of stochasticity in the transmission process is a systematic limitation of the proposed model. To capture the stochasticity of the transmission process, we must employ a stochastic process model to describe the transmission event, e.g., a branching process or a renewal process. Second, we did not explicitly use susceptibility of the exposed population, and also the background information on the traced contacts. While those datasets were not routinely collected, their use could help increase the validity of the forecast. Third, vaccination history of cases was not taken into consideration. Depending on residual immunity, we may observe a different clinical form of measles, i.e., modified measles. This could lead to a different (potentially longer) time delay from illness onset to diagnosis compared with the primary form of measles. Lastly, our assumptions included a fixed delay distribution function over the whole period of the outbreak. As we additionally verified, the inclusion of a step-like temporal dependence of the mean and variance of the delay function with given switching times (e.g. 29 March and/or 3 April as the dates of raised awareness 15) did not improve the model fit.

In conclusion, we demonstrated a simple generation-dependent model that was able to adequately capture an observed transmission pattern of the measles outbreak in Japan, 2018. The proposed model also helped predict the future incidence and evaluate public health control measures. Polishing the forecasting model further, we can achieve an eventual routine forecast and evaluation the outbreaks while maintaining the model structure as simple as possible.

## Competing Interests

The authors declare no competing interests.

## Data Availability

The code snippets used for simulations and generation of figures as well as the epidemiological count data are accessible from the GitHub repository: https://github.com/aakhmetz/MeaslesJapan2018.

## Corresponding Author

Hiroshi Nishiura (nishiurah@med.hokudai.ac.jp)

## Appendix

*A. Time-varying delay function*

Here we describe the fitting procedure when the delay distribution function *h* is a mixture of two distributions. The first distribution describes all cases whose times of illness onset are prior to a calendar time *τ*. In our exposition, the delay function *h* follows a Weibull distribution with parameters ***θ***_h_^(0)^ = {*μ*_*h*_^(0)^, *ν_h_*^(0)^}. The second distribution describes all cases with the time of illness onset later than a calendar time *τ*. It also follows a Weibull distribution with a set of parameters ***θ***_*h*_^(1)^ = {*μ*_*h*_^(1)^, *ν_h_*^(1)^}. The likelihood to describe the time delay from illness onset to laboratory confirmation is given by the formula:

\begin{equation*}L_{\text{delay}}\Bigl(\!\{\mathbf{\theta}_h^{(0,1)},\tau\};\,d_n,t_n\!\Bigr)=\prod\limits_{n=1}^N
                        h(d_n;\,\mathbf{\theta}_h^{(I(t_n-\tau))})\,,\end{equation*}

where *t_n_* is the time of illness onset for each particular case: *n* = {1 ... *N*}; *I*(*x*) is a step function: it equals to one when its argument *x* is non-negative, and zero otherwise. Analogously, we characterize the likelihood for new measles cases by the formula:

\begin{equation*}L_{\text{onset}}\Bigl(\!\{\mathbf{\theta}_\Lambda,\mathbf{\theta}_h^{(0,1)},\tau\};\,i_t\!\Bigr)
                        = {}\end{equation*}

\begin{equation*}\prod_{t=1}^T\frac{(H(T-t;\,\mathbf{\theta}_h^{(I(t-\tau))})\cdot\Lambda_t(\mathbf{\theta}_\Lambda))^{i_t}\exp({-}H(T-t;\mathbf{\theta}_h^{(I(t-\tau))})\cdot\Lambda_t(\mathbf{\theta}_\Lambda))}{i_t!}.\end{equation*}

Whereas, the total (composite) likelihood is given by a product of two likelihoods written above:

\begin{equation*}L_\Sigma\Bigl(\!\mathbf{\theta}=\{\mathbf{\theta}_\Lambda,\mathbf{\theta}_h^{(0,1)},\tau\};\,d_n,t_n,i_t\!\Bigr)={}\end{equation*} \begin{equation*}\qquad\qquad
                        L_{\mathrm{delay}}\Bigl(\!\{\mathbf{\theta}_h^{(0,1)},\tau\};d_n,
                        t_n\!\Bigr)\,
                        L_{\mathrm{onset}}\Bigl(\!\{\mathbf{\theta}_\Lambda,\mathbf{\theta}_h^{(0,1)}\};i_t\Bigr)\,.\end{equation*}

The total likelihood is maximized with respect to each parameter in the set ***θ***, consisting of (4 + *m*) parameters (*m* is the number of generations). Hence, the penalized likelihood used for model comparison based on AIC values can be defined as: 2(4 + *m* - ln *L_Σ_*(***θ***; *d_n_*, *t_n_, i_t_*)).

Model performance is shown in Figure 5 that can be compared with previous case of time-independent distribution *h* shown in Figure 2.

**Estimated parameter values and model comparison for a simple case of time-varied distribution of the delay h.**
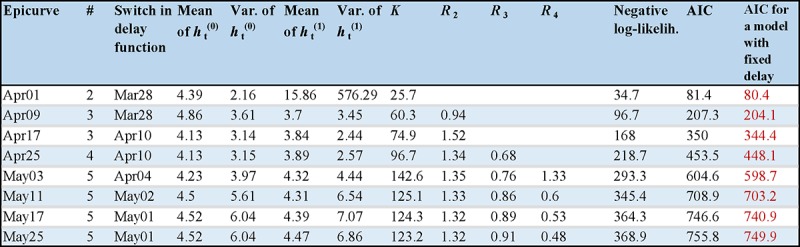
For any epicurve only the cases with minimal AIC values over a set of varied number of generations are shown. The switch in delay function indicates the optimal switching time, i.e., the calendar time on which the distribution is considered to have changed. The mean and variance of the delay distribution function before the switching day are indicated by the variable *h_t_*^(0)^, after the switching day by the variable *h_t_*^(1)^. The AIC values for a model with fixed distribution of the delay are shown in the last column, while the minimal AIC values are additionally indicated in red.

*B. Derivation of the generation-based model*

Our generation-dependent model rests on a well-known renewal equation, i.e.,

\begin{equation*}(\mathrm{B1})\qquad\qquad
                        i(t)=R(t)\int_0^\infty
                        i(t-s)g(s)\,ds\,,\end{equation*}

where *R*(*t*) represents the instantaneous reproduction number at calendar time *t*, *i*(*t*) is the incidence, and *g*(*s*) is the probability density function of the generation time of length *s*. For any *t* > 0, the density of incidence at time *t* is given by the generation expansion 16:

\begin{equation*}(\mathrm{B2})\qquad\qquad
                        i(t)=\sum_{m=0}^\infty
                    i_m(t),\end{equation*}

where *i_m_*(*t*) results from the iteration process:

\begin{equation*}(\mathrm{B3})\qquad\qquad
                        i_{m+1}(t)=R_m\int_0^\infty
                        i_m(t-s)g(s)\,ds\,.\end{equation*}

Here *R_m_* is the cohort-reproduction number of generation *m* (or "(*m *+ 1)-th" generation in the main text if we included the index case as generation 1). The integral of *i_m_*(*t*) over *t* gives the total size of generation *m*, and thus, *R_m_* can be mathematically interpreted as the asymptotic per-generation growth factor of the genealogy, consistent with the definition of the basic reproduction number 17.

Replacing the right-hand side of (B2) by that of (B3), we obtain Λ_*t*_ in the main text. As such, it should be noted that we perform forecasting by estimating the generation-dependent average number of secondary cases generated by a single primary case, which is interpreted as the cohort reproduction number (i.e., the average number of secondary cases generated by a primary case who was born at calendar time *t*), and not as the instantaneous reproduction number.
